# TLR7 Negatively Regulates B10 Cells Predominantly in an IFNγ Signaling Dependent Manner

**DOI:** 10.3389/fimmu.2020.01632

**Published:** 2020-07-28

**Authors:** Sathi Babu Chodisetti, Adam J. Fike, Phillip P. Domeier, Nicholas M. Choi, Chetna Soni, Ziaur S. M. Rahman

**Affiliations:** Department of Microbiology and Immunology, Pennsylvania State University College of Medicine, Hershey, PA, United States

**Keywords:** toll-like receptor 7, B10 cells, B regulatory cells, IFNGR, lupus, autoimmunity

## Abstract

IL-10 producing B cells (B10 cells) play an important immunoregulatory role in various autoimmune and infection conditions. However, the factors that regulate their development and maintenance are incompletely understood. Recently, we and others have established a requirement for TLR7 in promoting autoimmune antibody forming cell (AFC) and germinal center (GC) responses. Here we report an important additional role of TLR7 in the negative regulation of B10 cell development. TLR7 overexpression or overstimulation promoted the reduction of B10 cells whereas TLR7 deficiency rescued these cells in both non-autoimmune and autoimmune-prone mice. TLR7 expression was further inversely correlated with B cell-dependent IL-10 production and its inhibition of CD4 T cell proliferation and IFNγ production in an *in vitro* B cell and T cell co-culture system. Further, B10 cells displayed elevated TLR7, IFNγR, and STAT1 expression compared to non-B10 cells. Interestingly, deficiency of IFNγR in TLR7 overexpressing lupus-prone mice rescued B10 cells from TLR7-mediated reduction. Finally, B cell intrinsic deletion of IFNγR was sufficient to restore B10 cells in the spleens of TLR7-promoted autoimmune mouse model. In conclusion, our findings demonstrate a novel role for the IFNγR-STAT1 pathway in TLR7-mediated negative regulation of B10 cell development.

## Introduction

B cells generally mount anti-pathogen immune responses by producing antibodies, cytokines, and antigen presentation to T helper cells (1). However, a subset of B cells known as regulatory B cells (Bregs) negatively regulate cellular immune responses and inflammation (2). Bregs produce immunoregulatory cytokines such as interleukin 10 (IL-10), transforming growth factor β (TGF-β) and IL-35 to mediate immunosuppression (3–6). IL-10-producing Bregs were previously shown to negatively regulate T helper cell functions and promote the development of regulatory T cells (7). Several IL-10 producing Breg cell subsets such as transitional type 2- marginal-zone precursor (T2-MZP) cells, CD5^+^CD1d^hi^ B cells, Tim-1^+^ B cells, plasma cells, and plasmablasts have been reported in various infection and inflammation conditions (8–11). CD5^+^CD1d^hi^ B cells in the spleen that exclusively produce IL-10 (designated B10 cells), suppress both autoimmune and hypersensitivity responses (9, 12). Mice deficient in IL-10-producing B cells exhibit exacerbated experimental autoimmune encephalitis (4). Further, adoptive transfer of *ex vivo* expanded IL-10^+^ B cells markedly inhibited the disease symptoms in mice with established EAE (13) whereas adoptive transfer of IL-10-deficient B cells to autoimmune arthritic mice fails to suppress inflammation (7). Together, these reports highlight the importance of Breg or B10 cells in regulating immune responses.

A substantial number of previous studies indicated inflammation and autoimmune conditions to be the prerequisite for Breg or B10 cell differentiation (14). pDCs were shown to drive the differentiation of immature B cells into IL-10-producing B cells and plasmablasts through IFN-α production and CD40 co-stimulation (15). Gut microbiota-driven IL-1β and IL-6 were also shown to promote differentiation of IL-10-producing B cells in an arthritic mouse model (16). Several other proinflammatory cytokines such as IL-21, IL-35, GM-CSF, and IL-15 were also shown to promote Breg cell expansion under inflammatory conditions (13, 17, 18). In addition to the roles of pro-inflammatory cytokines in Breg or B10 cell differentiation, stimulation through B cell receptor (BCR), and CD40 was also shown to induce B cell derived IL-10 production (4). Furthermore, toll-like receptor (TLR) signaling such as TLR4-MyD88 signaling was shown to confer regulatory function to B cells that suppress Th1/Th17 responses and the disease in the EAE model (19). Although these previous studies have identified various factors including TLR4 in promoting Breg/B10 cell differentiation, the role of RNA sensing through TLR7 in regulating these cells remains unknown.

TLR7 is an endosomal receptor that recognizes microbial or self-antigen-derived single stranded RNA ligands (20). TLR7 is highly implicated in the development of SLE in which it recognizes RNA-containing immune complexes (21–23). Overexpression or overactivity of TLR7 promotes severe SLE disease in the mouse models (21) whereas TLR7 deficiency in B cells completely abrogates the disease symptoms (24–26). We also have recently shown the development of SLE-associated antibody forming cell (AFC) and germinal center (GC) responses by TLR7 overexpression or overstimulation, promoting the generation of autoreactive B cells and autoantibodies (27). However, whether TLR7 expression contributes to the differentiation and maintenance of IL-10 producing B cells in the context of SLE autoimmune response remains unknown. Further, during an autoimmune response, the inflammatory cytokine signals that govern the differentiation of B10 cells in the context of TLR7 overexpression remain to be elucidated during an autoimmune response.

Although both Type I and II interferon (IFN) signaling contribute to SLE development (28–30), we recently have reported an indispensable role for IFNγ signaling in TLR7-mediated development of autoimmunity (27). The importance of B cell intrinsic IFNγ signaling in the development of autoreactive B cells and autoantibody responses has also been described (27, 31, 32). However, the role of IFNγ signaling in cytokine-secreting B10 cells remains unknown. Here we used SLE mouse models with TLR7-sufficiency, -deficiency, -overexpression, and -overstimulation to dissect the roles of TLR7 and IFNγ signaling in the regulation of B10 cells. We found that TLR7 overexpression led to the reduction of B10 cells whereas TLR7 deficiency enhanced B10 cell frequency. TLR7 expression in B cells was inversely correlated with their IL-10 production capacity and IL-10 mediated inhibition of IFNγ production by CD4^+^ T cells. We observed that B10 cells expressed elevated levels of TLR7, IFNγR and STAT1 compared to other subsets of B cells. The observed TLR7 driven reduction of B10 cells was predominantly dependent on IFNγ signaling as decreased frequency of B10 cells in TLR7 overexpression models was rescued in the absence of IFNγR. Further, B cell specific deletion of IFNγR normalized the B10 cell frequency in TLR7 overexpression models. These results highlight the major role of B cell-intrinsic IFNγ signaling in the negative regulation of B10 cells in TLR7 promoted SLE.

## Materials and Methods

### Mice

C57BL/6J (B6), B6.129S7-*Ifngr1*^*tm*1*Agt*^/J (IFNγR1^−/−^), C57BL/6N-*Ifngr1*^*tm*1.1*Rds*^/J (IFNγR^fl/fl^), B6.SB-*Yaa/*J (B6^Yaa^), B6.Cg-Tg (TcraTcrb)425Cbn/J (OT-II–transgenic) mice were originally purchased from The Jackson Laboratory and bred in house. TLR7^−/−^ mice backcrossed to B6 mice for 10 generations were bred in-house. The B6.Sle1b (Sle1b) mice (congenic for the Sle1b sublocus) were described previously (33). B6.CD23^Cre^ (B6.Cg-Tg(Fcer2a-cre)5Mbu/J) mice, provided by Dr. Meinrad Busslinger were crossed to B6.Sle1b autoimmune mice in house. All animal studies were conducted at Penn State Hershey Medical Center in accordance with the guidelines approved by our Institutional Animal Care and Use Committee. Animals were housed in a specific pathogen free barrier facility.

### Imiquimod Treatment

For epicutaneous imiquimod treatment, 5% imiquimod cream (Glenmark Pharmaceuticals) was applied on the ears of mice, 3 times weekly for 8 weeks as previously described (27, 34).

### Cell Isolation and Flow Cytometry

Single cell suspensions were prepared from harvested spleens by mechanical disruption using frosted slides. Red blood cells were lysed with Tris Ammonium Chloride. Cells were stained with combinations of the following antibodies: B220-BV605 (RA3-6B2), CD19-BV605 (6D5), IL-10-PECy7 (JES5-16E3), Vβ2-AF700/APC (B20.1), IFNγ-APC (XMZ1.2), STAT1-PE (1/STAT1), CD23-biotin (B3H4), Streptavidin-PECy5, CD4-AF700 (RMP4-5), CD21-FITC (4E3), CD1d-PE (1B1), CD5-BV421/APC (53–7.3), TLR7-PE (A94B10), IFNγR-biotin (GR20), and Annexin V-APC. Cells were stained with fixable viability dye-eFluor780 (Invitrogen) prior to surface staining. For surface staining, cells were incubated with either fluorochrome conjugated Abs or biotinylated Abs followed by staining with streptavidin-fluorochrome conjugates. For IL-10 and IFNγ staining, cells were permeabilized according to manufacturer's instructions with the Cytofix and Cytoperm kit (BD Biosciences). TLR7 and STAT1 staining was performed using FoxP3 staining buffer kit (eBioscience) and phosphoflow staining kit (Perm buffer III, BD Biosciences), respectively. Stained cells were analyzed using the BD LSR II flow cytometer (BD Biosciences). Data were acquired using FACSDiva software (BD Biosciences) and analyzed using FlowJo software (Tree Star). For imaging flow cytometry, samples were analyzed on ImageStreamX equipped with 3 lasers using the following power set-up: 405 nm (120 mW) and 488 nm (200 mW). 60x magnification was used for image acquisition with 0.3 × 0.3 μm and field of view 40 × 170 μm. Data were analyzed by IDEAS 6.2 software using the application, spot count. Scale bar, 7 mm or 40× objective, for an overall magnification to 100× and 400×, respectively.

### *In vitro* Cell Cultures and Stimulations

B cells were purified from naïve 10–12 week old male or female mice with mouse anti-CD43 microbeads following the manufacturer's instructions (Miltenyi Biotec). Purified B cells or splenocytes were suspended (2 × 10^6^ cells/ml) in culture medium (RPMI-1640 containing 10% FCS, 200 μg/ml penicillin, 200 U/ml streptomycin, 4 mM L-glutamine, and 50 μM 2-ME) with LPS (10 μg/ml, *Escherichia coli* serotype 0111:B4; Sigma-Aldrich), PMA (50 ng/ml; Sigma-Aldrich), ionomycin (500 ng/ml; Sigma-Aldrich), and GolgiStop (BD Biosciences) for 5 h, in 24-well flat-bottom plates. For measuring IL-10 in culture supernatants, B cells were stimulated for 48 h with LPS (10 μg/ml), CD40 mAb (5 μg/ml; clone FGK4.5 -UCSF mAb Core), and anti-mouse IgM Ab (10 μg/ml; Jackson ImmunoResearch Laboratories). IL-10 concentration in the culture supernatants were measured by mouse IL-10 DuoSet ELISA as per the manufacturer's instructions (R&D). To make B cells as antigen presenting cells (APCs), purified B cells were stimulated with 10 μg/ml anti-CD40 and 1 μg/ml LPS for 48h. Thereafter, cells were washed and pulsed with 10 μg/ml OVA-peptide (Invivogen) for 6 h. OT-II T cells were purified by negative selection using Pan T cell isolation kit II (Miltenyi Biotec), labeled with 3 μM CFSE, and co-cultured with OVA-peptide loaded B cells for 72–96 h. OT-II T cell proliferation and IFNγ production were assessed by flow cytometry. To determine the IL-10 specific inhibition on CD4 T cells proliferation, we co-cultured activated B cells (10 μg/ml anti-CD40 and 1 μg/ml LPS for 48 h) with CD4 T cells that were plated in plate bound anti-CD3 and soluble anti-CD28 mAbs and measured the cell proliferation by CFSE dye dilution assay in presence or absence of IL-10 blocking mAb (clone-JES5-2A5 from BioLegend).

### IL-10^+^ B Cell (B10) Isolation and Quantification

IL-10 producing B cells were isolated and enumerated using regulatory B cell isolation kit as per the manufacturer's instructions (Miltenyi Biotec). Briefly, isolated splenocytes were enriched for B cells using regulatory B cell biotin-antibody cocktail and anti-biotin microbeads. Enriched B cells (2.5 × 10^6^ cells/ml) were stimulated with LPS (10 μg/ml), PMA (50 ng/ml), and ionomycin (500 ng/ml) for 5 h for IL-10 production. B10 cells were specifically labeled by regulatory B cell catch reagent and detection antibody (PE) and sorted by MACS using anti-PE microbeads. The frequency of B10 cells was determined by flow cytometric analysis.

### Statistical Analysis

*P*-values were calculated using unpaired, non-parametric, Mann–Whitney, Student's *t*-test (for comparison of two groups) or one-way or two-way ANOVA, with a follow-up Tukey multiple-comparison test (for comparison of more than two groups). ns, non-significant, ^*^P < 0.05, ^**^P < 0.01, ^***^P < 0.001, and ^****^P < 0.0001. GraphPad Prism 6 software was used for statistical analysis.

## Results

### TLR7 Negatively Regulates B10 Cells in Both Non-autoimmune and Autoimmune-Prone Mice

**Figure 1:**
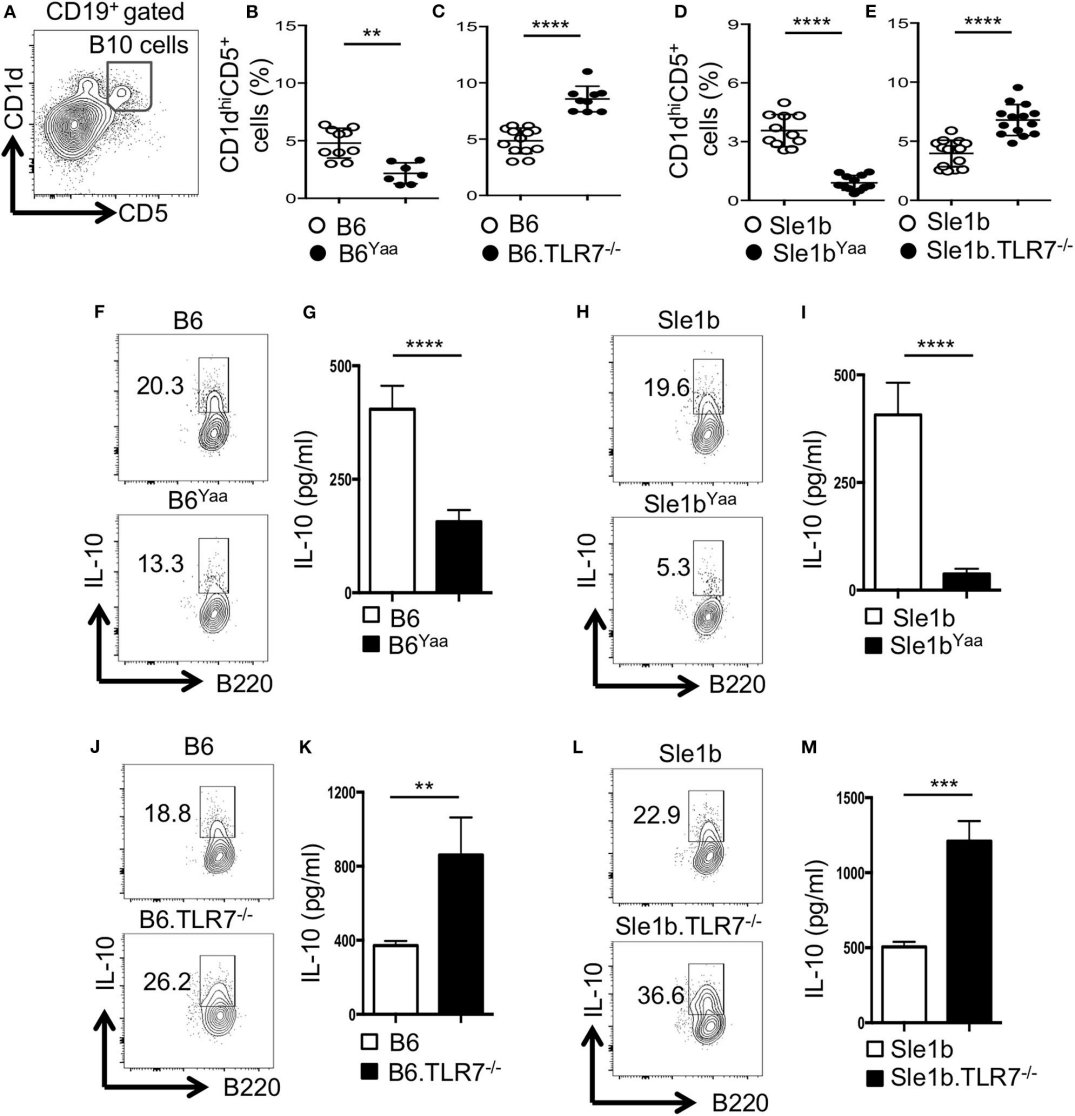
TLR7 expression negatively regulates IL-10 producing B cells in autoimmune and non-autoimmune mice. **(A–E)** Representative flow cytometric gating strategy **(A)** and the percentages **(B–E)** of splenic CD19^+^CD1d^hi^CD5^+^ B10 cells derived from male **(B,D)** or female **(C,E)** non-autoimmune B6 or autoimmune-prone Sle1b mice. **(F–M)** Purified B cells from B6 **(F,G,J,K)** or Sle1b **(H,I,L,M)** mice were stimulated with LPS and anti-CD40 mAb for 48 h. Cells were re-stimulated with PMA, Ionomycin and GolgiStop (monensin) cocktail for 5 h followed by intracellular staining for IL-10. **(F,H,J,L)** Representative flowcytometric contour plots show IL-10 expression in CD19^+^ gated population. **(G,I,K,M)** Secreted IL-10 was measured by ELISA from culture supernatants. The data shown are the cumulative results of 3 independent experiments. Statistical values were determined using an unpaired, non-parametric, Mann–Whitney, Student's *t*-test. **p < 0.01, ***p < 0.001, and ****p < 0.0001.

Previous studies identified CD19^+^CD1d^hi^CD5^+^ B cells (designated B10 cells) to be the major producers of IL-10 (9, 12). To determine the role of TLR7 in the development of B10 cells, we first analyzed non-autoimmune C57BL/6 (B6) mice deficient in TLR7 or B6 mice expressing an extra copy of TLR7 due to a translocation of the X chromosome to the Y chromosome (Y-chromosome autoimmune accelerator locus, Yaa). B6 mice carrying the Yaa locus (B6^Yaa^) had a reduced frequency of CD19^+^CD1d^hi^CD5^+^ B10 cells whereas B6 mice deficient in TLR7 (B6.TLR7^−/−^ mice) exhibited an increased percentage of these cells compared to B6 control mice (Figures 1A–C). To define the role of TLR7 in B10 cell development in an autoimmune setting, we crossed autoimmune prone B6.Sle1b (designated Sle1b) mice to the B6^Yaa^ and TLR7^−/−^ strains to generate Sle1b^Yaa^ and Sle1b.TLR7^−/−^ mice, respectively. We also found a significant reduction in B10 cell percentage in Sle1b^Yaa^ mice and conversely increased percentage of these cells in Sle1b.TLR7^−/−^ mice (Figures 1D,E). We observed CD19^+^CD1d^hi^CD5^+^ B cells to be the predominant producers of IL-10 compared to no or very low IL-10 production by CD1d^hi^CD5^−^ and CD1d^lo^CD5^−^ B cells in autoimmune-prone Sle1b mice (Figures S1A,B), confirming the published findings (12). Maximal IL-10 expression by CD1d^hi^CD5^+^ cells was further confirmed by the image stream analysis of CD19^+^CD1d^hi^CD5^+^ B10 and CD19^+^CD1d^lo^CD5^−^ non-B10 cells (Figure S1C).

LPS and CD40 signaling induce IL-10 production by B cells *in vitro* (12). Next, we examined the effects of TLR7 on the expression of IL-10 upon *in vitro* B cell stimulation by LPS and anti-CD40. Flow cytometric and ELISA data showed B cells derived from B6^Yaa^ and Sle1b^Yaa^ mice displayed significantly reduced IL-10 expression compared to their control counterparts and the differences were more pronounced in the autoimmune background (Figures 1F–I). Conversely, B cells from TLR7^−/−^ and Sle1b.TLR7^−/−^ mice showed an increase in IL-10 expression (Figures 1J–M). Together, these results suggest that TLR7 expression negatively affects the development of B10 cells.

### *In vivo* TLR7 Stimulation Reduces B10 Cell Frequency and Number

**Figure 2:**
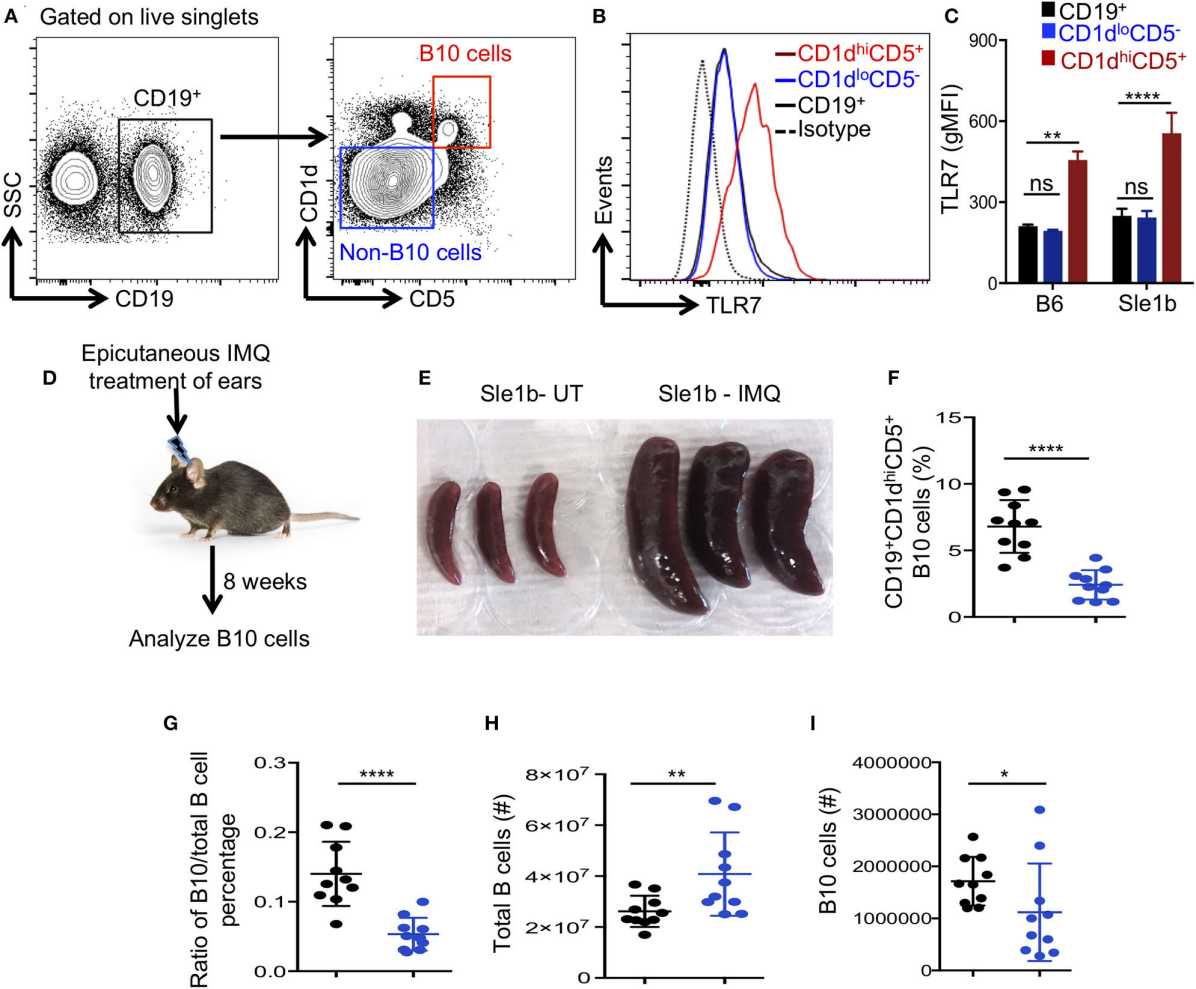
Imiquimod treatment induces depletion of B10 cells. **(A,B)** Representative flow cytometric gating strategy **(A)** and overlay of histograms **(B)** show the expression of TLR7 in CD19^+^ gated cell populations (CD19^+^-total B cells, CD1d^hi^CD5^+^-B10 cells, CD1d^lo^CD5^−^ non-B10 cells) that were derived from Sle1b mice. **(C)** The bar diagrams show geometric mean fluorescence of TLR7 in total B cell, B10 and non-B10 cell populations that were derived from B6 and Sle1b mice. **(D)** Schematic representation of epicutaneous IMQ treatment model. **(E)** Representative images of spleens that were treated or untreated (UT) with imiquimod (IMQ) for 8 weeks. The scatter plots represent the percentages of CD1d^hi^CD5^+^ B10 cells in CD19^+^ B cell gated population **(F)**, the ratio of percentages of CD19^+^CD1d^hi^CD5^+^ B10 cells and CD19^+^ total B cells in CD19^+^ B cell gated population **(G)**, total number of CD19^+^ B cells in lymphocyte gated population **(H)** and number of CD19^+^CD1d^hi^CD5^+^ B10 cells in CD19^+^ B cell gated population **(I)** that were quantified by flow cytometry in untreated (UT) and IMQ treated mice at 8 weeks time point. **(F–I)** Black and blue dots represent Sle1b-UT and Sle1b-IMQ mice, respectively. These data represent 2 independent experiments. Statistical values were determined using an unpaired, non-parametric, Mann–Whitney, Student's *t*-test. ns, non-significant, *p < 0.05, **p < 0.01, and ****p < 0.0001.

Next, we determined whether TLR7 expression differed among total B cells, CD1d^hi^CD5^+^ B10 cells and CD1d^lo^CD5^−^ non-B10 cells, which could explain the differential regulation of B10 and non-B10 cells by TLR7 signaling. Interestingly, B10 cells showed much higher TLR7 expression than non-B10 and total B cells (Figures 2A,B). This high TLR7 expression in B10 cells was observed in both non-autoimmune B6 and autoimmune-prone Sle1b mice (Figure 2C). We recently showed that epicutaneous application of imiquimod (IMQ), a synthetic TLR7 agonist, drives systemic autoimmunity by enhancing autoimmune GC and AFC responses (27). To further determine the relationship between TLR7 stimulation and the reduction of B10 cells *in vivo*, we treated mice with IMQ epicutaneously for 8 weeks as we and others previously described (Figure 2D). Similar to our recent report (27), IMQ induced splenomegaly in Sle1b mice (Figure 2E) and an expansion of total B cells within the spleen (Figure 2H), however, it resulted in a dramatic reduction in the frequency and number of splenic B10 cells (Figures 2F,I). Additionally, the ratio of percentage of B10 cells and total B cells was also reduced following IMQ treatment (Figure 2G). Together these data indicate that B10 cells express higher TLR7 and overstimulation of which reduces B10 cell numbers.

To determine the fate of B10 cells during TLR7 driven autoimmunity, we examined the viability and apoptosis of B10 and non-B10 cells in Sle1b, Sle1b^Yaa^, and Sle1b.TLR7^−/−^ mice at 3 months of age. We reasoned that the autoimmune responses would be initiated, but B10 cells would not be completely depleted at this time point, thus providing a window of time to assess viability. Interestingly, we found no differences in the frequency of non-B10 (Figures S2A–D) or B10 cells (Figures S2E–H) undergoing apoptosis or cell death among all three strains. These data indicate that the reduction of B10 cells during TLR7 mediated autoimmune responses is likely not due to cell death.

### B Cell Derived IL-10 Suppresses CD4^+^ T Cell Proliferation and IFNγ Expression in a TLR7 Dependent Manner

**Figure 3:**
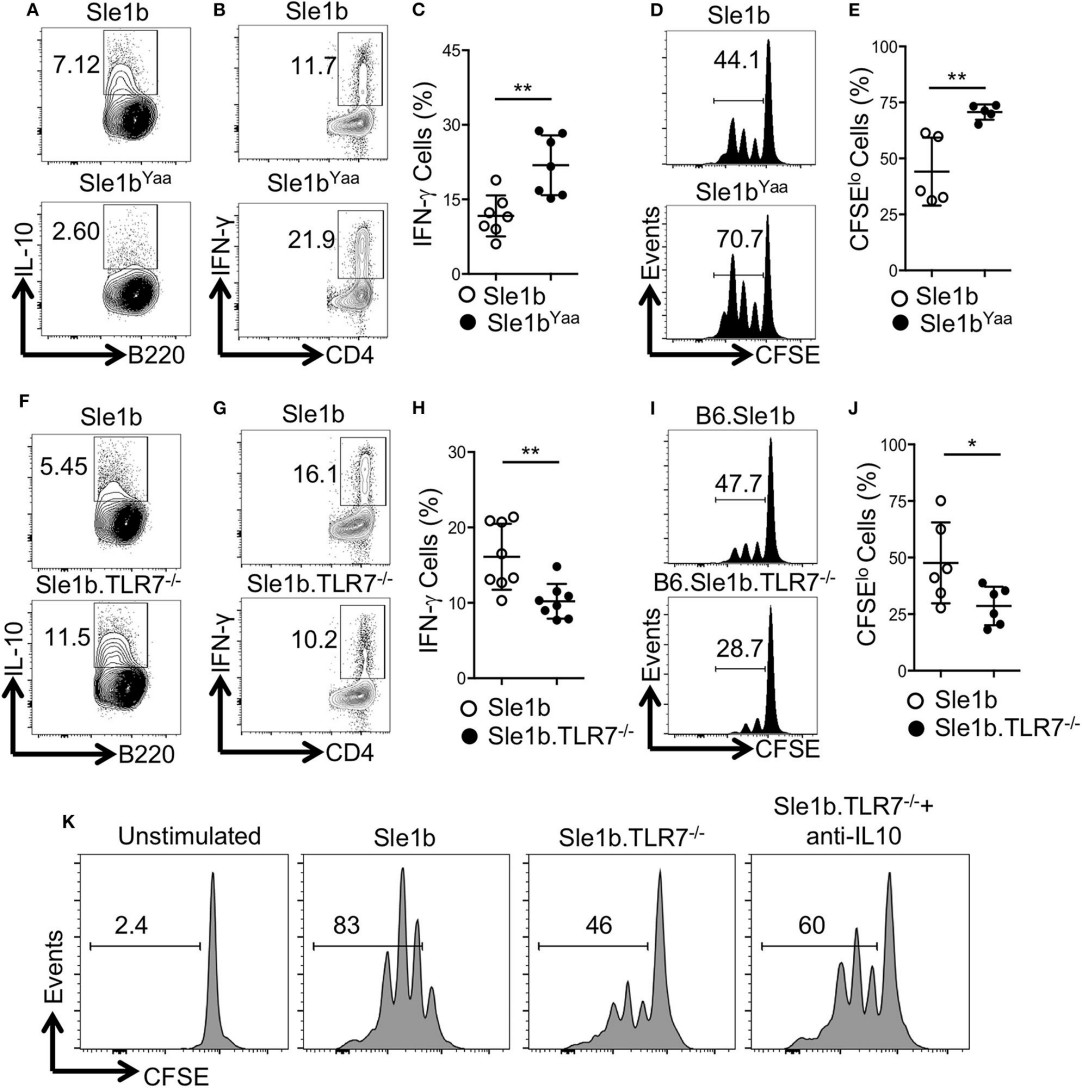
IL-10^+^ B cells inhibit antigen specific CD4 T cells in a TLR7-dependent manner. Purified B cells were isolated from indicated genotypes and activated with LPS and anti-CD40 mAb for 36 h. Cells were pulsed with 5 μg/ml OVA peptide (323–339) for overnight. Purified OT-II CD4 T cells were CFSE labeled and co-cultured with peptide primed B cells in presence of OVA peptide for 96 h. **(A,F)** Representative flow plots show IL-10 expression in B cells after 48 h of co-culture. Representative contour plots **(B,G)** and bar diagrams **(C,H)** display IFN-γ expression in co-cultured CD4 T cells. **(D,E,I,J)** Representative histogram plots **(D,I)** show CD4 T cell proliferation by CFSE dye dilutions assay and the bar diagrams **(E,J)** show percentages of CFSE^lo^ CD4 T cells after 72 h of co-culture. **(K)** Histograms represent proliferation profile of CD4^+^ T cell that were co-cultured with activated B cells from indicated mouse strains in presence or absence of IL-10 neutralizing mAb. The numbers in the plots show percentages of CFSE^lo^ cells. The data shown are the cumulative results of 2–3 independent experiments. Statistical values were determined using an unpaired, non-parametric, Mann–Whitney, Student's *t*-test. *p < 0.05 and **p < 0.01.

To determine how TLR7 expression in B cells may affect antigen specific CD4^+^ T cells, we co-cultured OVA specific OT-II CD4^+^ T cells with OVA-peptide pulsed B cells that were pre-stimulated with LPS and anti-CD40 mAb. Similar to data shown in Figure 1, TLR7 expression in B cells inversely correlated with IL-10 expression in the T and B cell co-culture system (Figures 1, 3A,F). Moreover, B cell derived IL-10 production in the co-culture system also inversely correlated with intracellular IFNγ expression by OT-II T cells (Figures 3B,C,G,H). Further, we observed an increased proliferation of OT-II T cells co-cultured with TLR7 overexpressing B cells that had lower capacity to produce IL-10 compared to decreased proliferation of OT-II T cells co-cultured with TLR7-deficient B cells with higher IL-10 production capacity (Figures 3D,E,I,J). Finally, the decreased proliferation of CD4^+^ T cells that were co-cultured with B cells from Sle1b.TLR7^−/−^ mice was modestly reversed in the presence of IL-10 neutralizing mAb (Figure 3K). These results indicate that B cell derived IL-10 inhibits proliferation and IFNγ expression by co-cultured CD4^+^ T cells in a TLR7 dependent manner.

### B10 Cells Express Increased Levels of IFNγR and STAT1

**Figure 4:**
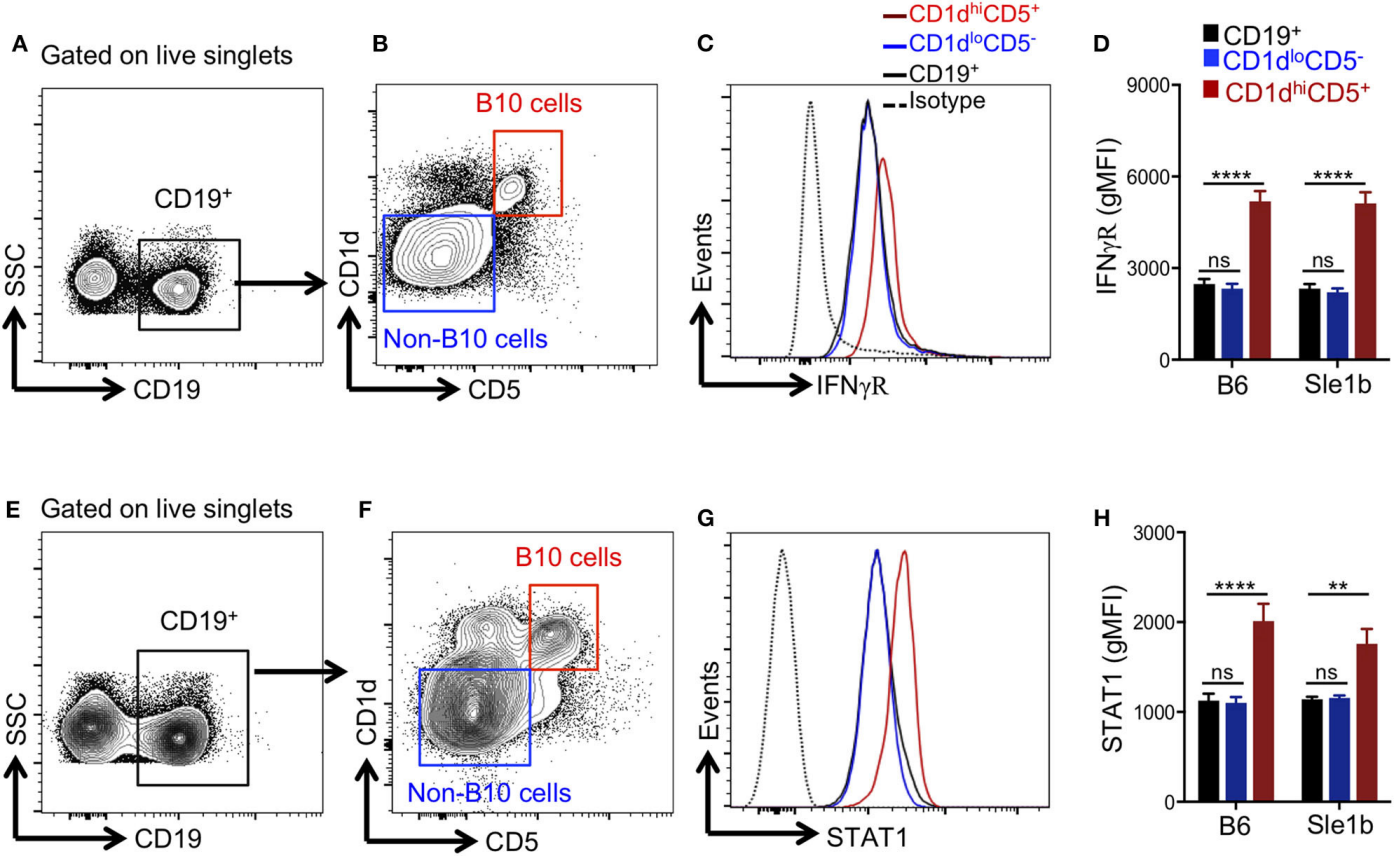
B10 cells show higher STAT1 and IFNγR expression. Representative flow cytometric gating strategy **(A,B,E,F)** and overlay histograms **(C,G)** show expression of IFNγR **(C)**, and total STAT1 **(G)** in indicated cell populations derived from Sle1b mice. The bar diagrams show geometric mean fluorescence of IFNγR **(D)** and STAT1 **(H)** in CD19^+^ total B cells, CD1d^hi^CD5^+^ B10 cells, CD1d^lo^CD5^−^ non-B10 cells from B6 and Sle1b mice. These data represent 2 independent experiments. Statistical analysis was performed by one-way ANOVA, with a follow-up Tukey multiple-comparison test. ns, non-significant, **p < 0.01, and ****p < 0.0001.

Several pro-inflammatory cytokines have been described to promote Breg/B10 cell differentiation (13, 15, 16), yet the role of IFNγ signaling in the development of these cells was previously not explored. Recently, we have reported an essential role of IFNγ signaling in the development of spontaneous (31) and TLR7 driven (27) autoimmune responses. We therefore asked whether IFNγ signaling was involved in the depletion of B10 cells. First, we determined the expression levels of IFNγR in total B cells, CD1d^hi^CD5^+^ B10 cells and CD1d^lo^CD5^−^ non-B10 cells and observed elevated levels of IFNγR on B10 cells than non-B10 and total B cells (Figures 4A–C). Increased IFNγR expression in B10 cells was observed both in non-autoimmune B6 and autoimmune-prone Sle1b mice (Figure 4D). In support of this data, we also observed an enhanced expression of STAT1, a transcription factor that functions downstream of IFNγR signaling, in B10 cells compared to total B cells or CD1d^lo^CD5^−^ non-B10 cells (Figures 4E–H). These results suggest that an increased IFNγR-STAT1 signaling in B cells may be involved in the depletion of B10 cells.

### TLR7 Mediates Reduction of B10 Cells Predominantly in an IFNγ Signaling Dependent Manner

**Figure 5:**
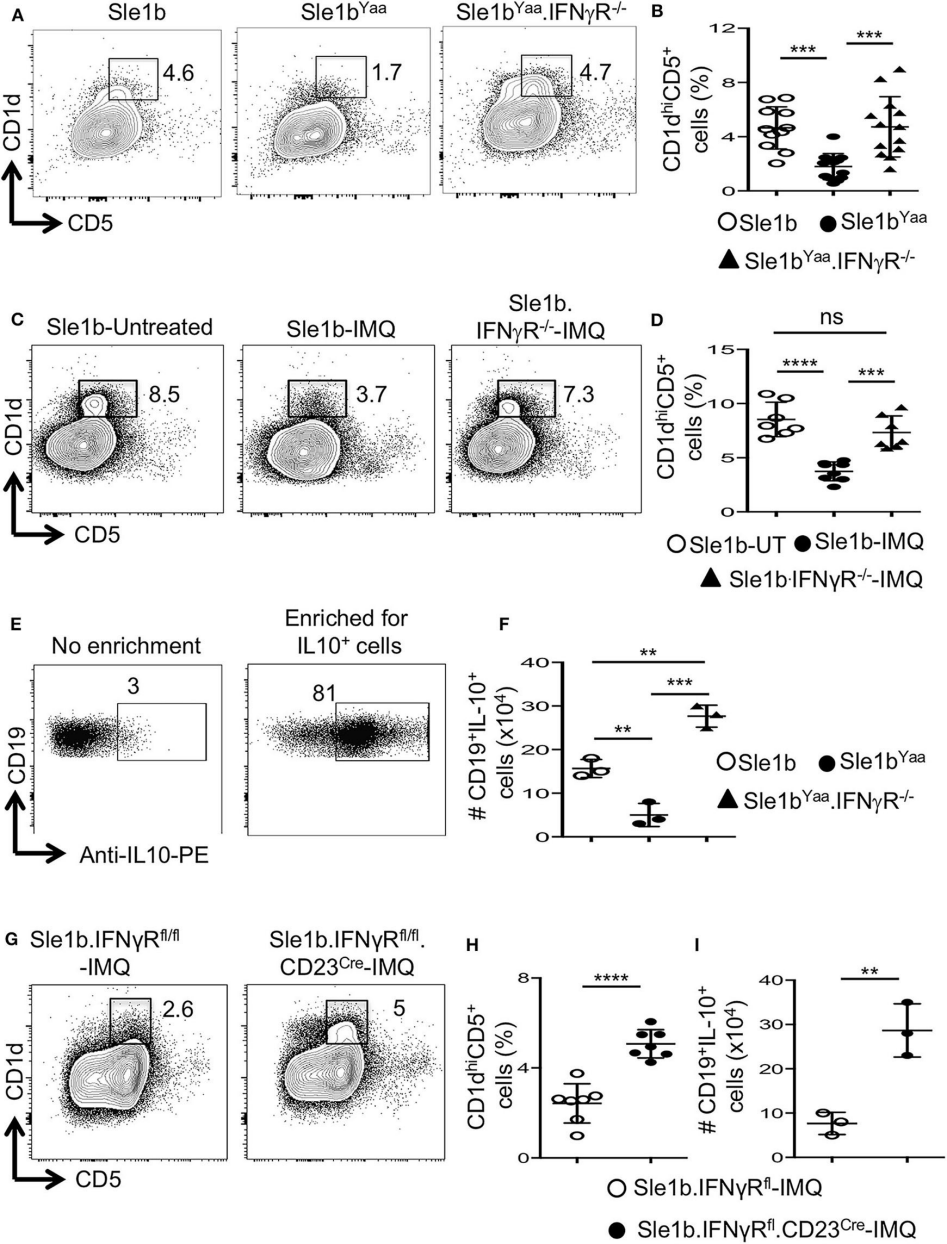
TLR7 medicated depletion of B10 cells is dependent on IFNγR signaling. Representative flow plots **(A)** show CD19^+^CD1d^hi^CD5^+^ B10 cells in indicated mouse strains at the age of 6 mo. Scatterplot **(B)** shows percentage of B10 cells as gated in **(A)**. Representative flow plots **(C)** and percentage **(D)** of splenic CD19^+^CD1d^hi^CD5^+^ cells in 8-week IMQ treated mice. **(E,F)** Splenocytes from indicated mouse stains were stimulated *in vitro* for the enrichment of B10 cells using mouse B regulatory cell isolation kit as described in the methods. Representative flow plots **(E)** show the efficiency of CD19^+^IL-10^+^ B cell enrichment. Scatter plot **(F)** depicts the number of CD19^+^IL-10^+^ B cells quantified by flow cytometry in splenocytes from indicated mice. Representative flow plots **(G)** and percentage **(H)** of splenic CD19^+^CD1d^hi^CD5^+^ B10 cells in imiquimod (IMQ) treated mice of indicated genotypes. **(I)** Scatter plot depicts the number of CD19^+^IL-10^+^ B cells in spleen that were enriched by mouse B regulatory cell isolation kit and quantified by flow cytometry. Data represent 3 experiments. Statistical analysis was performed by one-way ANOVA, with a follow-up Tukey multiple-comparison test **(B,D,F)** or unpaired, non-parametric, Mann–Whitney, Student's *t*-test **(H,I)**. ns, non-significant, **p < 0.01, ***p < 0.001, and ****p < 0.0001.

Considering the possible involvement of IFNγR-STAT1 signaling in TLR7-mediated reduction of B10 cells, we crossed autoimmune-prone B6.Sle1b.Yaa (designated Sle1b^Yaa^) mice with IFNγR deficient mice to generate Sle1b^Yaa^.IFNγR^−/−^ mice. We found that CD19^+^CD1d^hi^CD5^+^ B10 cells were significantly reduced in Sle1b^Yaa^ mice whereas IFNγR deficiency fully rescued these cells (Figures 5A,B). TLR7-mediated reduction of B10 cells upon imiquimod treatment of Sle1b mice were also salvaged by an IFNγR deficiency (Figures 5C,D). IFNγR deficiency also led to an increased number of IL-10^+^ B cells in Sle1b^Yaa^ mice that were enumerated using a B regulatory cell isolation kit in a flow cytometry analysis (Figures 5E,F). These results demonstrate a major role of IFNγR signaling in the reduction of B10 cells in TLR7 driven autoimmune mice.

To determine whether IFNγR signaling in B cells is required for the reduction of B10 cells, we conditionally deleted *Ifn*γ*r1* gene in peripheral B cells in B6.Sle1b mice by crossing with IFNγR^fl/fl^ and CD23^Cre^ mice (designated Sle1b.IFNγR^fl/fl^.CD23^Cre+^ mice). All B cells including B10 cells were deficient of the *Ifn*γ*r1* gene in Sle1b.IFNgR^fl/fl^.CD23^Cre+^ mice (data not shown). Deletion of the *Ifn*γ*r1* gene in B cells significantly replenished the frequency of B10 cells in Sle1b.IFNγR^fl/fl^.CD23^Cre+^ mice treated with IMQ (Figures 5G,H). Concurrently, B cell intrinsic IFNγR deficiency also led to an increased number of IL-10^+^ B cells enumerated using the B regulatory cell isolation kit and flow cytometry (Figure 5I). These data indicate a B cell intrinsic role of IFNγ signaling in TLR7-driven reduction of B10 cells.

## Discussion

Although various inflammatory cytokines (16–18) and toll-like receptor (TLR) signals (19) were previously described to regulate IL-10-producing Breg or B10 cells, the role of RNA sensing through TLR7 in the development of these regulatory B cells is unknown. In this study, using TLR7-deficient and TLR7-overexpression or -overactivation mouse models we discovered that TLR7 negatively regulates the development of B10 cells. TLR7 overactivity resulted in a significant reduction of B10 cells whereas TLR7-deficiency rescued these cells. We further observed that TLR7 promoted reduction of B10 cells predominantly in an IFNγ signaling dependent manner.

We and others previously demonstrated the B cell intrinsic role of TLR7 in the development of autoimmune germinal centers (24, 25). In line with these findings, we recently reported that TLR7 overactivity induces SLE-like autoimmunity in mice by augmenting autoimmune antibody-forming cell (AFC) and GC responses (27). Our current data show that TLR7 overexpression or overstimulation significantly reduced the frequency and number of B10 cells and their IL-10 producing capacity. Our data suggest a differential regulation of GC and B10 cells by TLR7, presumably through varied levels of TLR7 expression in these cells. Our published and current data together indicate that TLR7 promotes SLE pathogenesis by positively and negatively regulating GC and B10 cell development, respectively. Given elevated AFC and GC responses upon TLR7 overexpression (27) we, however, cannot rule out the role of non-B10 cell responses in TLR7-mediated negative regulation of B 10 cells. Although there is a strong correlation between elevated autoimmune AFC and GC responses, and significantly reduced frequency of B10 cells and their IL-10 production, currently we do not have the data to conclude whether loss of B10 cells upon TLR7 overexpression or overstimulation contribute to heightened AFC and GC responses in TLR7-driven SLE-autoimmunity. It is also not clear whether significantly reduced B10 cells caused by TLR7 overactivity play any role in promoting SLE-like autoimmune disease in these mice.

TLR signaling has previously been implicated in the development of Breg/B10 cells. TLR4-MyD88 signaling was shown to drive the regulatory function of B cells that suppressed Th1 and Th17 cell responses and the disease in the EAE model (19). Prolonged exposure of B cells to the TLR4 ligand, LPS, *in vitro* also lead to an increased frequency of IL-10 producing CD1d^hi^CD5^+^ B10 cells (12). Further, TLR9 dependent recognition of self-DNA derived from apoptotic cells was shown to induce IL-10 production by both mouse and human B cells (35). TLR9 was also shown to play a role in the expansion of B10 cells induced by sublethal total body irradiation of mice (36). In contrast, others have noted that B cells stimulated with TLR9 ligands resulted in a reduction of B10 cells when BCR specificity was restricted (2). Our current *in vivo* data show loss of B10 cells upon TLR7 overexpression or overstimulation. Overall, earlier findings paired with our current results indicate that levels of TLR signaling either positively or negatively impact B10 cell development. Future studies will focus on understanding the dichotomy between different TLR stimulation signals and the induction of IL-10 and how this could be exploited for the treatment of autoimmunity.

Although both type I and type II interferons are associated with SLE (28–30), we recently showed an indispensable role of IFNγ signaling in TLR7-promoted development of autoreactive B cells and systemic autoimmunity (27). TLR7 stimulation enhanced the IFNγ receptor expression on B cells and induced T cell derived IFNγ expression *in vivo* (27), suggesting TLR7-mediated activation of IFNγ signaling in B cells. Consistent with this notion we have demonstrated the essential role of B cell intrinsic IFNγ signaling in autoreactive B cell development in TLR7-promoted SLE-autoimmunity (27). However, the regulation of B10 cell development in this autoimmune model by IFNγ signaling remained unknown. In this study, using a TLR7 driven model of SLE with a B cell specific deletion of IFNγR, we report an important B cell-intrinsic role of IFNγ signaling in the reduction of B10 cells. These data suggest a differential regulation of autoreactive B cell and B10 cell development by IFNγ signaling in B cells in TLR7-promoted autoimmunity. Based on our published data showing a non-redundant role of IFNγ signaling in elevated AFC and GC responses (27), the effects of IFNγ signaling on B10 cell development can be direct in a cell-intrinsic manner or indirect in which non-B10 cells can promote IFNγ production by Th1 or Tfh cells that, in turn, regulates B10 cells via IFNγR signaling. Multiple studies have documented that persistent inflammatory conditions result in decreased B10 cell populations, notably most of these studies were performed in immunocompetent hosts with an intact T cell compartment (2, 37, 38). Given our published data showing CD4^+^ T cells to be the major producers of IFNγ upon treatment of mice with TLR7 ligand (27), persistent IFNγ signaling in B cells may contribute to the decrease in B10 cells in other inflammatory systems.

We recently have demonstrated that the B cell intrinsic deletion of IFNγR signaling in a TLR7 driven autoimmune system results in severe reduction in antinuclear antibodies and immune complex deposition within the kidney (27). Given our current findings in this study and the role for B10 cells in the negative regulation of autoimmune responses, it is interesting to speculate that one mechanism by which IFNγR signaling in B cells contributes to systemic autoimmunity in TLR7-driven SLE mice is through controlling the development of B10 cells. While TLR7 deficiency enhanced the frequency of B10 cells in Sle1b.TLR7^−/−^ mice, we found IFNγR deficiency did not enhance, but rescued the loss of B10 cells, caused by TLR7 overexpression or overstimulation, back to wild type level. These data suggest that additional mechanisms may contribute to B10 cell loss in TLR7-driven autoimmunity. Type I IFN and IL21 signaling that play significant roles in SLE development, which were induced in IMQ treated Sle1b mice (27), are most likely additional mechanisms involved in TLR7-mediated negative regulation of B10 cells.

It remains unclear whether the loss of B10 cells in TLR7 overexpression or overstimulation autoimmune systems resulted from B10 cells differentiating into IL-10 producing plasma cells or undergoing apoptosis. A large proportion of B10 cells are believed to be derived from marginal zone (MZ) B cells, and overstimulation of MZ B cells with a TLR4 ligand results in activation-induced cell death (39). We and others previously demonstrated that overexpression or over stimulation of TLR7 resulted in a significant decrease in MZ B cells (21, 34), and an increase in GC B cell and AFC populations in autoimmune prone mice (27). Therefore, an increase in B10 cell death by the persistent or overstimulation of TLR7 could explain the reduction in B10 cell frequency and number in TLR7 driven SLE mice. However, we found no difference in the frequency of B10 cells undergoing apoptosis or cell death, indicating that the reduction of B10 cells during TLR7 mediated autoimmune responses is likely not due to cell death. Although previous studies indicated inflammation and autoimmune conditions to be the driving force for Breg or B10 cell expansion (14, 40), it is not clear why the inflammatory environment in TLR7 overexpression or stimulation SLE-prone mice did not similarly expand B10 cell populations. To date, little is known about the differentiation trajectory of B10 cells under TLR7-promoted autoimmune condition as no lineage tracing experiments have been performed. Therefore, we are currently unable to conclude at which stage in B10 differentiation TLR7 stimulation may have the largest effects.

In summary, we have uncovered a previously unrecognized roles of TLR7 and IFNγ signaling in the regulation of B10 cells. Although we cannot rule out the role of non-B10 cells in TLR7-mediated negative regulation of B10 cells, given significantly increased levels of TLR7, IFNγR, and STAT1 expression in B10 cells we propose that TLR7 and IFNγ signaling in B10 cells synergistically decrease their numbers, thereby promoting autoimmune responses. Future studies focusing on delineating the mechanisms by which TLR7 and IFNγ signaling may synergize to negatively regulate B10 cell development are warranted. In conjunction with our previous observations concerning the roles of IFNγ and TLR7 in the regulation of SLE, our current findings reinforce the importance of potentially targeting these pathways for the treatment of SLE.

## Data Availability Statement

All datasets presented in this study are included in the article/Supplementary Material.

## Ethics Statement

All animal studies were conducted at Penn State Hershey Medical Center in accordance with the guidelines approved by our Institutional Animal Care and Use Committee.

## Author Contributions

SC and ZR designed experiments. SC performed most of the experiments. AF, PD, NC, and CS performed specific experiments. SC, AF, and ZR wrote the manuscript. All authors contributed to the article and approved the submitted version.

## Conflict of Interest

The authors declare that the research was conducted in the absence of any commercial or financial relationships that could be construed as a potential conflict of interest.

## References

[1] LeBien TW Tedder TF. B lymphocytes: how they develop and function. Blood. (2008) 112:1570–80. 10.1182/blood-2008-02-078071 18725575 PMC2518873

[2] Rosser EC Mauri C. Regulatory B cells: origin, phenotype, and function. Immunity. (2015) 42:607–12. 10.1016/j.immuni.2015.04.005 25902480

[3] Shen P Roch T Lampropoulou V O'Connor RA Stervbo U Hilgenberg E. IL-35-producing B cells are critical regulators of immunity during autoimmune and infectious diseases. Nature. (2014) 507:366–70. 10.1038/nature12979 24572363 PMC4260166

[4] Fillatreau S Sweenie CH McGeachy MJ Gray D Anderton SM. B cells regulate autoimmunity by provision of IL-10. Nat Immunol. (2002) 3:944–50. 10.1038/ni833 12244307

[5] Parekh VV Prasad DV Banerjee PP Joshi BN Kumar A Mishra GC. B cells activated by lipopolysaccharide, but not by anti-Ig and anti-CD40 antibody, induce anergy in CD8+ T cells: role of TGF-beta 1. J Immunol. (2003) 170:5897–911. 10.4049/jimmunol.170.12.5897 12794116

[6] Tian J Zekzer D Hanssen L Lu Y Olcott A Kaufman DL. Lipopolysaccharide-activated B cells down-regulate Th1 immunity and prevent autoimmune diabetes in nonobese diabetic mice. J Immunol. (2001) 167:1081–9. 10.4049/jimmunol.167.2.1081 11441119

[7] Carter NA Vasconcellos R Rosser EC Tulone C Muñoz-Suano A Kamanaka M. Mice lacking endogenous IL-10-producing regulatory B cells develop exacerbated disease and present with an increased frequency of Th1/Th17 but a decrease in regulatory T cells. J Immunol. (2011) 186:5569–79. 10.4049/jimmunol.1100284 21464089

[8] Evans JG Chavez-Rueda KA Eddaoudi A Meyer-Bahlburg A Rawlings DJ Ehrenstein MR. Novel suppressive function of transitional 2 B cells in experimental arthritis. J Immunol. (2007) 178:7868–78. 10.4049/jimmunol.178.12.7868 17548625

[9] Yanaba K Bouaziz JD Haas KM Poe JC Fujimoto M Tedder TF. A regulatory B cell subset with a unique CD1dhiCD5+ phenotype controls T cell-dependent inflammatory responses. Immunity. (2008) 28:639–50. 10.1016/j.immuni.2008.03.017 18482568

[10] Ding Q Yeung M Camirand G Zeng Q Akiba H Yagita H. Regulatory B cells are identified by expression of TIM-1 and can be induced through TIM-1 ligation to promote tolerance in mice. J Clin Invest. (2011) 121:3645–56. 10.1172/JCI46274 21821911 PMC3163958

[11] Matsumoto M Baba A Yokota T Nishikawa H Ohkawa Y Kayama H. Interleukin-10-producing plasmablasts exert regulatory function in autoimmune inflammation. Immunity. (2014) 41:1040–51. 10.1016/j.immuni.2014.10.016 25484301

[12] Yanaba K Bouaziz JD Matsushita T Tsubata T Tedder TF. The development and function of regulatory B cells expressing IL-10 (B10 cells) requires antigen receptor diversity and TLR signals. J Immunol. (2009) 182:7459–72. 10.4049/jimmunol.0900270 19494269 PMC3733128

[13] Yoshizaki A Miyagaki T DiLillo DJ Matsushita T Horikawa M Kountikov EI. Regulatory B cells control T-cell autoimmunity through IL-21-dependent cognate interactions. Nature. (2012) 491:264–8. 10.1038/nature11501 23064231 PMC3493692

[14] Vitale G Mion F Pucillo C. Regulatory B cells: evidence, developmental origin and population diversity. Mol Immunol. (2010) 48:1–8. 10.1016/j.molimm.2010.09.010 20950861

[15] Menon M Blair PA Isenberg DA Mauri C. A regulatory feedback between plasmacytoid dendritic cells and regulatory B cells is aberrant in systemic lupus Erythematosus. Immunity. (2016) 44:683–97. 10.1016/j.immuni.2016.02.012 26968426 PMC4803914

[16] Rosser EC Oleinika K Tonon S Doyle R Bosma A Carter NA. Regulatory B cells are induced by gut microbiota-driven interleukin-1β and interleukin-6 production. Nat Med. (2014) 20:1334–9. 10.1038/nm.3680 25326801

[17] Wang RX Yu CR Dambuza IM Mahdi RM Dolinska MB Sergeev YV. Interleukin-35 induces regulatory B cells that suppress autoimmune disease. Nat Med. (2014) 20:633–41. 10.1038/nm.3554 24743305 PMC4048323

[18] Rafei M Hsieh J Zehntner S Li M Forner K Birman E. A granulocyte-macrophage colony-stimulating factor and interleukin-15 fusokine induces a regulatory B cell population with immune suppressive properties. Nat Med. (2009) 15:1038–45. 10.1038/nm.2003 19668193

[19] Lampropoulou V Hoehlig K Roch T Neves P Calderón Gómez E Sweenie CH. TLR-activated B cells suppress T cell-mediated autoimmunity. J Immunol. (2008) 180:4763–73. 10.4049/jimmunol.180.7.4763 18354200

[20] Petes C Odoardi N Gee K. The toll for trafficking: toll-like receptor 7 delivery to the endosome. Front Immunol. (2017) 8:1075. 10.3389/fimmu.2017.01075 28928743 PMC5591332

[21] Deane JA Pisitkun P Barrett RS Feigenbaum L Town T Ward JM. Control of toll-like receptor 7 expression is essential to restrict autoimmunity and dendritic cell proliferation. Immunity. (2007) 27:801–10. 10.1016/j.immuni.2007.09.009 17997333 PMC2706502

[22] Subramanian S Tus K Li QZ Wang A Tian XH Zhou J. A Tlr7 translocation accelerates systemic autoimmunity in murine lupus. Proc Natl Acad Sci USA. (2006) 103:9970–5. 10.1073/pnas.0603912103 16777955 PMC1502563

[23] Fairhurst AM Hwang SH Wang A Tian XH Boudreaux C Zhou XJ. Yaa autoimmune phenotypes are conferred by overexpression of TLR7. Eur J Immunol. (2008) 38:1971–8. 10.1002/eji.200838138 18521959 PMC2993003

[24] Soni C Wong EB Domeier PP Khan TN Satoh T Akira S. B cell-intrinsic TLR7 signaling is essential for the development of spontaneous germinal centers. J Immunol. (2014) 193:4400–14. 10.4049/jimmunol.1401720 25252960 PMC4201954

[25] Jackson SW Scharping NE Kolhatkar NS Khim S Schwartz MA Li QZ. Opposing impact of B cell-intrinsic TLR7 and TLR9 signals on autoantibody repertoire and systemic inflammation. J Immunol. (2014) 192:4525–32. 10.4049/jimmunol.1400098 24711620 PMC4041708

[26] Christensen SR Shupe J Nickerson K Kashgarian M Flavell RA Shlomchik MJ. Toll-like receptor 7 and TLR9 dictate autoantibody specificity and have opposing inflammatory and regulatory roles in a murine model of lupus. Immunity. (2006) 25:417–28. 10.1016/j.immuni.2006.07.013 16973389

[27] Chodisetti SB Fike AJ Domeier PP Singh H Choi NM Corradetti C. Type II but not Type I IFN signaling is indispensable for TLR7-promoted development of autoreactive B cells and systemic autoimmunity. J Immunol. (2020) 48:1–8. 10.4049/jimmunol.1901175 31900342 PMC7002260

[28] Pollard KM Cauvi DM Toomey CB Morris KV Kono DH. Interferon-γ and systemic autoimmunity. Discov Med. (2013) 16:123–31. 23998448 PMC3934799

[29] Theofilopoulos AN Baccala R Beutler B Kono DH. Type I interferons (alpha/beta) in immunity and autoimmunity. Annu Rev Immunol. (2005) 23:307–36. 10.1146/annurev.immunol.23.021704.115843 15771573

[30] Crow MK. Type I interferon in the pathogenesis of lupus. J Immunol. (2014) 192:5459–68. 10.4049/jimmunol.1002795 24907379 PMC4083591

[31] Domeier PP Chodisetti SB Soni C Schell SL Elias MJ Wong EB. IFN-γ receptor and STAT1 signaling in B cells are central to spontaneous germinal center formation and autoimmunity. J Exp Med. (2016) 213:715–32. 10.1084/jem.20151722 27069112 PMC4854731

[32] Jackson SW Jacobs HM Arkatkar T Dam EM Scharping NE Kolhatkar NS. B cell IFN-γ receptor signaling promotes autoimmune germinal centers via cell-intrinsic induction of BCL-6. J Exp Med. (2016) 213:733–50. 10.1084/jem.20151724 27069113 PMC4854732

[33] Morel L Croker BP Blenman KR Mohan C Huang G Gilkeson G. Genetic reconstitution of systemic lupus erythematosus immunopathology with polycongenic murine strains. Proc Natl Acad Sci USA. (2000) 97:6670–5. 10.1073/pnas.97.12.6670 10841565 PMC18697

[34] Yokogawa M Takaishi M Nakajima K Kamijima R Fujimoto C Kataoka S. Epicutaneous application of toll-like receptor 7 agonists leads to systemic autoimmunity in wild-type mice: a new model of systemic Lupus erythematosus. Arthritis Rheumatol. (2014) 66:694–706. 10.1002/art.38298 24574230

[35] Miles K Heaney J Sibinska Z Salter D Savill J Gray D. A tolerogenic role for Toll-like receptor 9 is revealed by B-cell interaction with DNA complexes expressed on apoptotic cells. Proc Natl Acad Sci USA. (2012) 109:887–92. 10.1073/pnas.1109173109 22207622 PMC3271931

[36] Hong J Fang J Lan R Tan Q Tian Y Zhang M. TLR9 mediated regulatory B10 cell amplification following sub-total body irradiation: implications in attenuating EAE. Mol Immunol. (2017) 83:52–61. 10.1016/j.molimm.2017.01.011 28110075

[37] Lykken JM Candando KM Tedder TF. Regulatory B10 cell development and function. Int Immunol. (2015) 27:471–7. 10.1093/intimm/dxv046 26254185 PMC4817073

[38] Gorosito Serrán M Fiocca Vernengo F Beccaria CG Acosta Rodriguez EV Montes CL Gruppi A. The regulatory role of B cells in autoimmunity, infections and cancer: perspectives beyond IL10 production. FEBS Lett. (2015) 589:3362–9. 10.1016/j.febslet.2015.08.048 26424657

[39] Figgett WA Fairfax K Vincent FB Le Page MA Katik I Deliyanti D. The TACI receptor regulates T-cell-independent marginal zone B cell responses through innate activation-induced cell death. Immunity. (2013) 39:573–83. 10.1016/j.immuni.2013.05.019 24012421

[40] Mauri C Ehrenstein MR. The ‘short’ history of regulatory B cells. Trends Immunol. (2008) 29:34–40. 10.1016/j.it.2007.10.004 18289504

