# Supplementary material for: TLR7 Negatively Regulates B10 Cells Predominantly in an IFNγ Signaling Dependent Manner
Source: Front Immunol. 2020 Jul 28;11:1632. doi: 10.3389/fimmu.2020.01632 (PMC7399053; doi:10.3389/fimmu.2020.01632)
Supplement: Supplementary file 1 [file Data_Sheet_1.PDF]

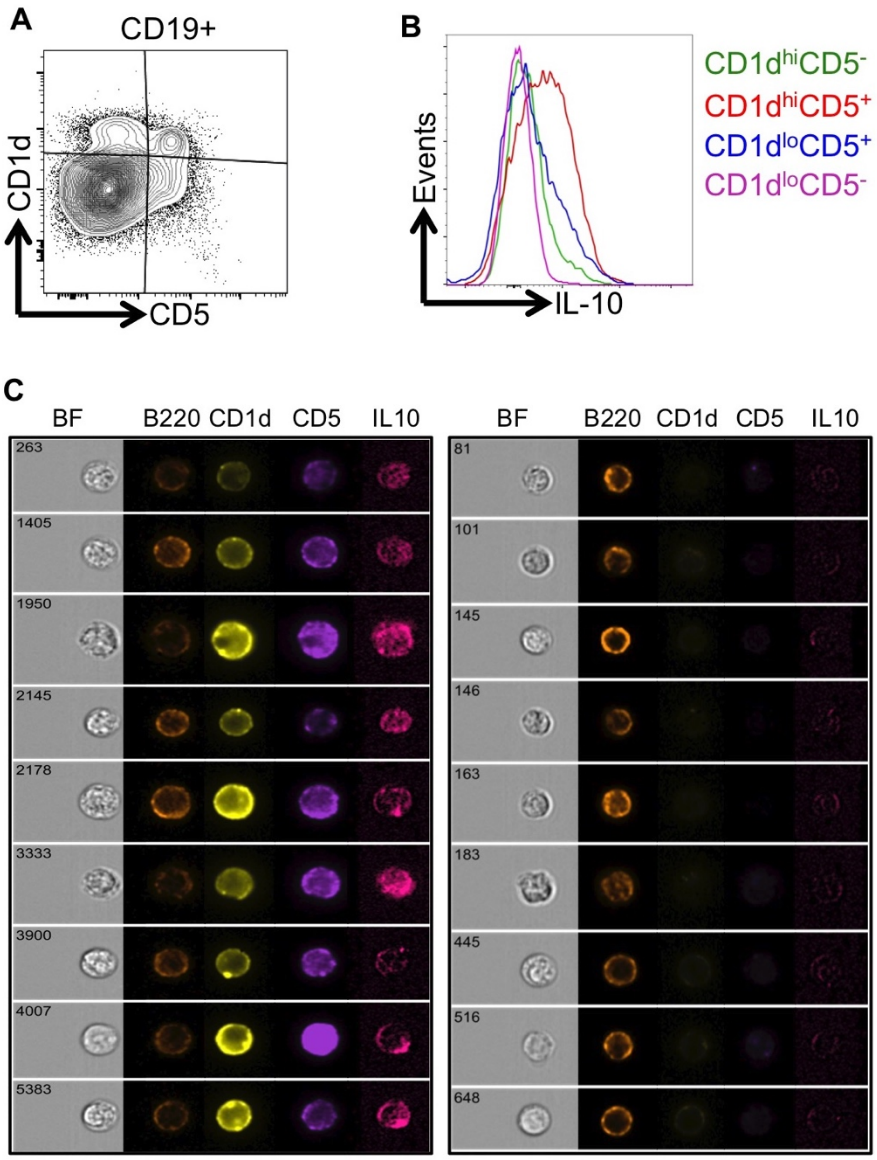

FIGURE S1: CD1d^{hi}CD5^{+} B cells are predominant IL-10 producers in Sle1b autoimmune mouse model. Splenocytes derived from Sle1b mice were cultured with LPS, PMA, Ionomycin and Monensin for 5 hrs and stained for CD19, CD1d and CD5 followed by intracellular staining for IL-10. Representative contour plot (**A**) show-gating strategy for CD1d^{hi}CD5^{-}, CD1d^{hi}CD5^{+}, CD1d^{lo}CD5^{+}, and CD1d^{lo}CD5^{-} cells. Flow cytometric overlay histogram (**B**) shows IL-10 expression in the indicated cell populations. (**C**) Splenocytes from Sle1b mice were stimulated as described in A, B and stained for IL-10 for the Imagestream analysis. Representative Imagestream images for B220^{+}CD1d^{hi}CD5^{+} B10 cells and B220^{+}CD1d^{lo}CD5^{-} non-B10 cells.

Figure S2

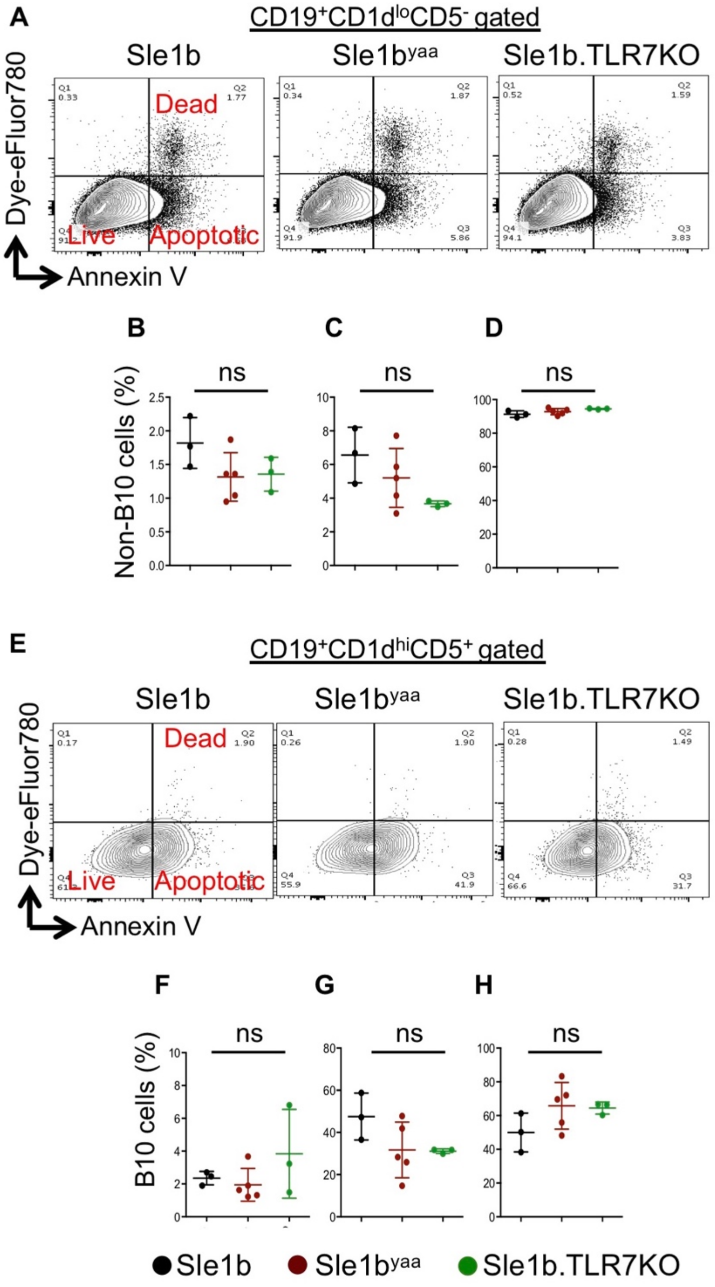

Figure S2: TLR7 expression has not effect on the spontaneous cell death of B10 and non-B10 cells. (**A, E**) Representative flow plots show live, apoptotic and dead cell populations of gated cells in the spleens of indicated mouse strains at the age of 3 mo. Scatterplots show percentages of dead (**B, F**), apoptotic (**C, G**) and live (**D, H**) cell populations in non-B10 ($CD19^{+}CD1d^{lo}CD5^{-}$) and B10 ($CD19^{+}CD1d^{hi}CD5^{+}$) cell fractions. Data represent a single experiment. Statistical analysis was performed by one-way ANOVA with a follow-up Tukey multiple-comparison. ns, non-significant.
